# Geographical, clinical, clinicopathological and radiographic features of canine angiostrongylosis in Irish dogs: a retrospective study

**DOI:** 10.1186/2046-0481-65-5

**Published:** 2012-03-20

**Authors:** Barbara Gallagher, Sheila F Brennan, Micaela Zarelli, Carmel T Mooney

**Affiliations:** 1 University Veterinary Hospital, UCD, Belfield, Dublin 4, Ireland

**Keywords:** Angiostrongylosis, *A. vasorum*, Dogs, Coagulopathy

## Abstract

**Background:**

*Angiostrongylus vasorum infection *is associated with high morbidity and mortality in dogs. Although recognised in Ireland, there are no large series of cases reported. The aim of this retrospective study was to identify pertinent clinical and geographical features in Irish dogs.

**Results:**

The case records of dogs presenting to the University College Dublin Veterinary Hospital (1999-2010) were reviewed. A contemporaneous review of external faecal parasitology and post mortem submissions was also performed. A positive diagnosis of angiostrogylosis was identified in 49 dogs including 24 clinical, 10 post mortem and 15 external faecal sample cases. The majority (n = 44 (90%)) resided on the East Coast.

In the clinical cases, the median age was 20 months, 29% of cases were older than 2 years. Clinical features included cardiorespiratory (63%), coagulopathic (71%) and other (63%) signs. Cough (n = 10), dyspnoea (n = 5) and tachypnoea (n = 3) were the most common cardiorespiratory abnormalities. Of animals with evidence of coagulopathy, excessive haemorrhage from a wound (n = 5), airway haemorrhage (n = 9), epistaxis (n = 3), haematoma (n = 4), suspected haemarthrosis (n = 3), neurological signs (n = 2) and haematuria (n = 1) were found. Ten dogs were anaemic, of which two were severe (haematocrit ≤ 0.20 L/L). Ten animals had thrombocytopenia, with four severely affected (≤50 × 10^9^/L). PT and APTT values were prolonged in 4 (24%) of 17 and a BMBT was prolonged in 5 (63%) of 8 cases. Vague signs of exercise intolerance (n = 6), lethargy (n = 6) and weakness (n = 2) were identified, with two (8%) animals having only these signs. In one animal the diagnosis appeared to be incidental. Thoracic radiographs (n = 19) identified abnormalities in 100% of cases. Four (17%) animals died before or within 24 hours of treatment and post mortem examinations confirmed angiostrongylosis. Fenbendazole was administered in 19 cases, 18 (95%) recovered. Two animals were euthanised, one which failed to respond to therapy and another in which an ante mortem diagnosis had not been made.

**Conclusions:**

Angiostrongylosis is not uncommon in Ireland, is not confined to young dogs or the East Coast and can present with a wide variety of signs, particularly coagulopathic, respiratory or neurological signs.

## Introduction

*Angiostrongylus vasorum *is a nematode with an indirect life cycle, belonging to the superfamily Metastrongyloidea. Primary hosts include wild and domestic dogs, in which the adult parasitises the right heart and pulmonary arteries. Following sexual reproduction ova are released into the pulmonary circulation, maturing into L1 larvae which emerge in the alveoli. L1 larvae enter the gastrointestinal tract after they are expelled from the trachea by coughing, they are swallowed and later excreted in the faeces. Intermediate hosts, such as aquatic and terrestrial snails or slugs [1] consume the larvae, providing a new host for further larval development. Paratenic hosts, such as the common frog *(Rana temporaria) *have been described in the life cycle and they can also act as intermediate hosts [2]. Patent infection is seen experimentally in dogs between 49 and 60 days after ingestion of intermediate hosts [1].

*A. vasorum *was first reported in south west France, and has since been recognised in many other countries worldwide including south east England and Wales, Ireland, Germany, Denmark, Canada, and south America [1,3-11]. Recently there has been an increase in the number of reports of sporadic cases from countries and areas previously considered devoid of infection, particularly in the northern half of the United Kingdom [12,13]. In data collated from referral centers, likely presented with more complex and severe cases, there is an approximate mortality of 24% [7,8,10].

Although *A. vasorum *is considered endemic in Ireland, and anecdotally is confined to the East Coast, there is no large case series published to date. The aim of this study was to provide data on the geographic location, variety of clinical signs, clinicopathological and radiographic features, and the response to treatment and outcome in infected dogs from Ireland.

## Materials and methods

The medical records of all dogs presenting to the University College Dublin (UCD) Veterinary Hospital between July 1999 and December 2010 were retrospectively reviewed for a positive diagnosis of *A. vasorum*. Reviewing the same time period, the computer records of the parasitology and pathology departments were interrogated using key words (*Angiostrongylus vasorum*, angiostrongylosis, *A. vasorum *and L1 larvae) for positive faecal and post mortem diagnoses from external cases, to provide additional information on geographic location and to limit the inherent geographic bias of the referred clinical cases. The files from each clinical case were examined for data on geographical location, signalment, presenting history and physical examination findings. Diagnostic tests, including clinicopathological analyses, radiographic features, coagulation abnormalities and other specific tests if performed were collated. Five cases have been reported previously, although in a small preliminary case series or highlighting other specific aspects not addressed in this paper [14-16].

Blood samples were obtained by jugular or cephalic venepucture and collected in ethylenediaminetetraacetic acid (EDTA) for haematological analyses using either the CELL-DYN 3500 (Abbott Laboratories) or ADVIA 2120 (Siemens Healthcare Diagnostics) systems. Samples were also placed in lithium heparin tubes for subsequent biochemical analyses using the Randox Daytona (Randox) or Randox RX Imola (Randox) systems. If requested sodium citrated samples collected for prothrombin time (PT) and activated partial thromboplastin time (APTT) were assessed using the KC4 Amelung (Amelung). D-dimers were measured using the Minutex D-dimer (Biopool), while a routine Simplate bleeding time device (Organon Teknika) was used to quantify the buccal mucosal bleeding time (BMBT). Diagnostic images were retrospectively reviewed by a resident in diagnostic imaging under the supervision of a European Diplomate in Diagnostic Imaging using a grade system previously described [17].

Where faecal analysis was carried out, a modified Baermann technique was used by suspending a minimum of 5 g of faeces wrapped in gauze in a funnel of warm water. The solution was decanted 12 hours later, and the sample was centrifuged and then the supernatant was discarded and the sediment examined. Zinc sulphate floatation was performed by adding 330 g of zinc sulphate to 1000 ml of water followed by the addition and mixing of faeces. The less dense material then floated to the top and the solution was strained and centrifuged. A coverslip was applied to the top of the centrifuge tube allowing microscopic examination of the material in contact with the coverslip [18]. If required, faecal PCR was performed at Bristol University as previously described [19].

Bronchoalveolar lavage was carried out under general anaesthesia by infusing a sterile 0.9% sodium chloride solution into the bronchi via a lavage catheter. The fluid was immediately aspirated and samples submitted for cytological analysis and culture. Samples for cytology were centrifuged and the sediment was examined microscopically for the presence of L1 larvae with morphological characteristics indicative of *A. vasorum *[1].

Follow-up telephone conversations were carried out with all contactable clients that had a clinical case referred to the UVH. Information was collected on the type of parasite control administered prior to referral, possibility of ingestion of slugs/snails, known or presumed contact with foxes, affected litter mates and any recurrence of clinical signs.

## Results

Angiostrongylosis was identified in 24 clinical cases and 25 external submissions (15 faecal samples and 10 post mortem examinations). This represented 0.17% of all new case referrals (n = 13,998), 1.6% of faecal (n = 940) and 2.6% of post mortem (n = 388) submissions over the same time period.

**Figure 1:**
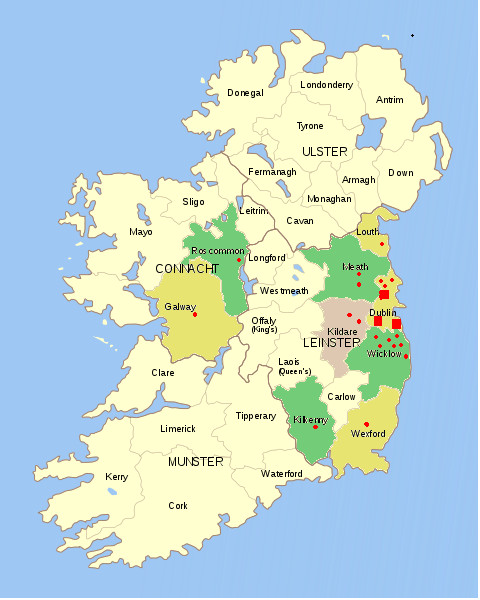
**Geographical location of all cases of angiostrongyloisis in clinical, faecal and post-mortem submissions to UCD Veterinary Hospital between 1999 and 2010**. "red circle"- 1 case "red square"- 10 cases.

The majority of dogs (n = 44 (90%)) resided on the East Coast of Ireland. Of the remaining cases there were two from county Kildare and a single case each from counties Kilkenny, Galway and Roscommon (Figure 1).

Of the 24 clinical cases there were nine (37.5%) males and 15 (62.5%) females. The median age was 20 (range 4-144) months. The group comprised four cross breeds and 20 pedigree dogs including German Shepherd (n = 3), Labrador Retriever (n = 2), Jack Russell Terrier (n = 2), Cocker Spaniel (n = 2), and one each of a variety of other breeds. From the telephone survey owners reported that foxes were known to frequent the garden to which the animal had access in 16 of 21 (76%) cases. Eight of 23 (35%) dogs were previously observed eating intermediate hosts. In four cases, another dog in the same household was affected. Two of these dogs died whilst undergoing investigation, as did their only respective housemate prior to referral.

Clinical signs included a variety of cardiorespiratory (n = 15 (63%)), coagulopathic (n = 17 (71%)) and other (n = 15 (63%)) less specific signs as presented in Table 1. Of the animals with clinical evidence of a primary or secondary coagulation defect, nine had either evidence of airway haemorrhage for which there was clinical evidence in five cases (haemoptysis) and diagnostic (radiographic (alveolar pattern) or cytological (ongoing or previous haemorrhage in BAL)) evidence in four. Neurological signs were noted in two animals and were considered to be secondary to intracranial haemorrhage which was confirmed in one case. The first animal presented with acute onset lethargy that progressed over 24 hours to stupor, hyperexcitability, anisocoria, non-responsive mydriatic pupils and eventually coma. The second animal presented with lethargy, acute onset collapse, unilateral hemiparesis and decreased menace bilaterally. Three animals presenting with lameness had suspected haemarthrosis and one animal presenting primarily for abdominal pain had a retroperitoneal haematoma. Vague clinical signs including exercise intolerance, lethargy, weakness and failure to thrive were also identified and were the only clinical signs in two animals.

**Table 1:** Clinical signs reported in 24 animals diagnosed with angiostrongylosis

Cardiorespiratory	n (%)	Coagulopathic	n (%)	Miscellaneous	n (%)
**Cough**	10(42)	Clinical evidence of pulmonary haemorrhage	5 (21)	Exercise intolerance	6 (25)

**Dyspnoea**	5 (21)	Diagnostic tests supporting pulmonary haemorrhage	4 (17)	Lethargy	6 (25)

**Tachypnoea**	3 (12)	Haemorrhage from wound	4 (17)	Weakness	2 (8)

**Nasal discharge**	1 (4)	Haematoma	4 (17)	Abdominal pain	1 (4)

**Heart murmur**	1 (4)	Epistaxis	3 (13)	Failure to thrive	1 (4)

**Syncope**	1 (4)	Suspected haemarthrosis	3 (13)	Lameness	3 (13)

**Sneezing**	1 (4)	CNS signs suspected secondary to haemorrhage	2 (8)		

Pneumothorax*	1 (4)	Bruising/ecchymoses	2 (8)		

		Haematuria	1 (4)		

*Developed five days post treatment, *n *number

Neutrophilia, anaemia and thrombocytopenia were the most common haematological abnormalities identified as presented in Table 2. Two cases were severely anaemic (haematocrit < 0.20 L/L). Eosinophilia was present in only three cases. The median eosinophil count was within the reference interval (0.3 × 10^9^/L (0-1.47 × 10^9^/L)) but individual values varied considerably (0-8.28 × 10^9^/L) (Table 2). Biochemical abnormalities tended to be mild and included increases in creatine kinase (n = 18 (75%)) concentration and amylase (n = 17 (71%)), alkaline phosphatase (n = 16 (67%)) and alanine aminotransferase (n = 12 (50%)) activities. Hyperglobulinaemia (n = 10 (42%)), hypoalbuminaemia (n = 10 (42%)) and hyperphosphataemia (n = 8 (33%)) were also reported. Mild hypercalcaemia (3.14 mmol/L and 3.16 mmol/L (2.3-3.0 mmol/L)) was noted in two (8%) cases. Corresponding phosphate values (0.8 mmol/L and 2.2 mmol/L) were within and above the reference interval (0.8-1.8 mmol/L), respectively.

**Table 2:** Haematological abnormalities from 24 cases of angiostrongyslosis

Parameter (Reference interval)	Median	Minimum	Maximum	% above reference interval	% below reference interval
**Haematocrit** **(0.37 - 0.55 L/L)**	0.38	0.13	0.63	4	42

**Thrombocyte count** **(150 - 500 **× **10^9^/L)**	164.5	2	619	8	42

**Neutrophil count** **3 - 11.5 **× **10^9^/L**	12.25	4.76	27.24	54	0

**Eosinophil count** **(0 - 1.47 **× **10^9^/L)**	0.3	0	8.28	13	0

The PT and APTT were assessed in 18 animals, which included 15 of the 17 cases with evidence of coagulopathy (Table 3). Of the four (27%) cases with prolonged values, two had reference interval BMBT and thrombocyte count, while the remaining animals each had either severe thrombocytopenia or a prolonged BMBT. A BMBT was performed in 8 of the coagulopathic animals and was prolonged in 5 (63%) cases. Three of these had reference interval PT/APTT and thrombocyte count and one each had either markedly prolonged PT and APTT or moderate thrombocytopenia (57 × 10^9^/L). Thrombocytopenia was severe enough to induce coagulopathy (≤ 50 × 10^9^/L) in four of ten animals and only one had additionally a mild prolongation in PT and APTT. In total, a reasonable explanation for the coagulopathic signs was evident in 11 animals. Two of the remaining six, despite presenting with significant haemorrhage had reference interval PT, APTT and BMBT, with only one having a thrombocyte count below the reference interval (98 × 10^9^/L) but above the value typically associated with overt haemorrhage. The remaining four had adequate thrombocyte counts but a BMBT and/or PT and APTT were not performed. In the seven cases without a documented clinical coagulopathy the thrombocyte count was within reference interval in all but one that was mildly decreased (148 × 10^9^/L). In four animals BMBT, PT and APTT were not performed. In the remaining animals, BMBT (n = 2) PT and APTT (n = 3) were within reference interval.

**Table 3:** Coagulopathic test results in dogs with haemorrhagic diathesis associated with angiostrongylosis. Values in red are abnormal

Case	BMBT (< 5 m)	Thrombocyte **(150 - 500 **× **10^9^/L)**	PT (7 - 14 s)	APTT (15 - 25 s)	Clinical signs
**1**	ND	116	9.8	14.2	Haematuria, Retroperitoneal haematoma

**2**	ND	7	18	26	Haemoptysis

**3**	> 5	57	8	14	Haemorrhage post surgery & from a broken nail

**4**	> 5	205	9.7	14.8	Suspected haemarthrosis (severe anaemia and lameness)

**5**	> 8	175	22.3	250	Haemorrhage from tongue

**6**	< 5	158	11.8	22.5	Haemoptysis

**7**	ND	2	10	9.7	Haemoptysis, Haemorrhage from mouth, Ecchymoses

**8**	< 5	98	10.7	14.7	Epistaxis, Haematoma, Suspected haemarthrosis

**9**	< 5	22	7.7	13.4	Epistaxis, Suspected CNS haemorrhage (multifocal CNS signs)

**10**	ND	145	7.2	14.1	BAL cytology suggested intrapulmonary haemorrhage

**11**	ND	258	ND	ND	BAL cytology suggested intrapulmonary haemorrhage

**12**	ND	178	10.3	27.8	BAL cytology suggested intrapulmonary haemorrhage

**13**	ND	199	28.5	28.2	Haemorrhage from lip, haemoptysis, Suspected haemarthrosis

**14**	ND	2	8.1	13.4	Radiographs suggestive of intrapulmonary haemorrhage

**15**	> 5	171	10.4	14.5	Haematoma, Bruising

**16**	ND	553	ND	ND	Unilateral epistaxis

**17**	> 5	155	11.7	16.1	Pulmonary haemorrhage, Multifocal intracranial haemorrhage& haematoma

*BMBT *buccal mucosal bleeding time

*m *minutes

*PT *prothrombin time

*s *seconds

*APTT *activated partial thromboplastin time

*ND *not determined

*BAL *Brochoalveolar lavage

**Figure 2:**
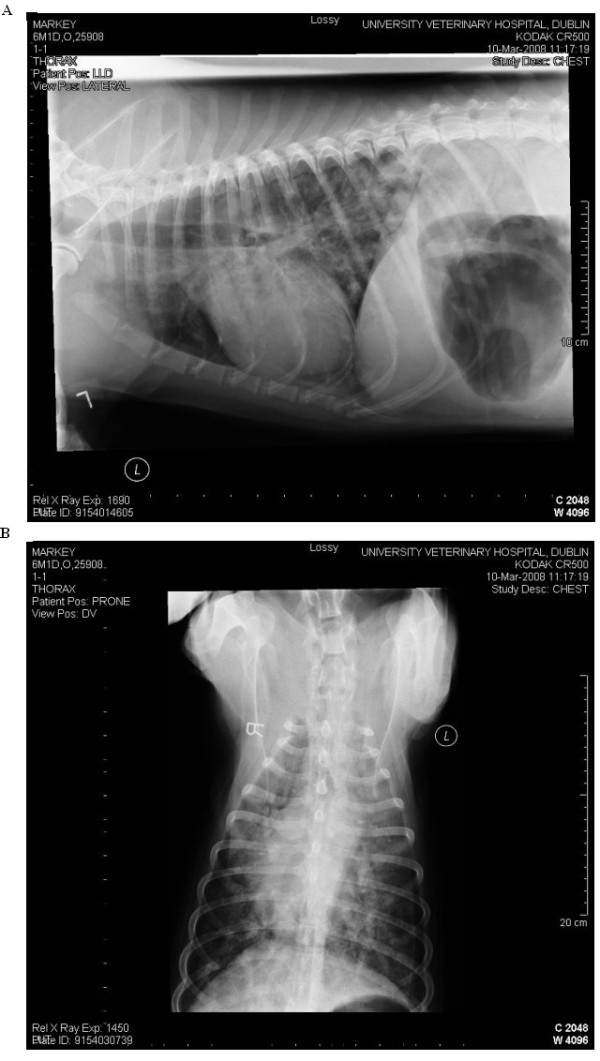
**Mixed pulmonary patterns on a dorsoventral and lateral view**.

Thoracic radiography was performed in 22 cases, nineteen of which were available for retrospective review. The results are presented in Table 4. Twelve animals had mixed patterns, including bronchoalveolar and interstitial (n = 5) (Figure 2), bronchoalveolar alone (n = 6) and alveolar-interstitial (n = 1) were also reported. While most patterns were described as diffuse, a bronchial pattern was localised to the left hemi-thorax in one case and alveolar patterns were localised in one case each to the cranial thorax, the left hemi-thorax and the periphery. Five (of 8) cases with pleural fissures had a concurrent moderate to severe diffuse reticular or hazy interstitial pattern. A pneumothorax developed five days after a bulla was observed in one case, which was being treated with fenbendazole, prednisolone and doxycycline. Repeat radiographs were available in six cases that completed a full course of anthelminthic treatment. All continued to have radiographic abnormalities despite a full clinical recovery and five of six that were tested demonstrating negative faecal Baermann flotations for a median of 49 (range 1-203) days post treatment.

**Table 4:** Radiographic features of 19 animals with angiostrongylosis

Radiographic feature	Cases affected	Median grade	Location		Other
				
			Diffuse	Localised	
**Bronchial pattern**	11	1	10	1	

**Interstitial pattern**	15	2	15	0	Hazy n = 9 Reticular n = 7 Nodular n = 1

**Alveolar pattern**	10	2	6	3	

**Vertebral heart score increased**	7	11.2			

**Right heart enlargement**	9	1			Majority right ventricular

**Pleural fissure**	10				

**Pleural effusion**	1	1			Small volume

**Bulla**	1				Progressed to pneumothorax

The grading system has been previously described [17]

Of the clinical cases, a diagnosis of angiostrongylosis was achieved by modified Baermann in 16 (72%) of 22 cases. In one of these animals a final diagnosis was made by modified Baermann 10 months after numerous negative faecals and two negative BALs. Negative results were obtained in six (28%) cases and a subsequent diagnosis was returned by post mortem (n = 4), BAL (n = 1) and faecal PCR (n = 1). Five (83%) of six cases in which a BAL was performed had negative results, in four of these modified Baermann was also negative. Whilst BAL was only positive in one case, three others had cytological evidence supporting previous haemorrhage. Faecal PCR was performed in one case, confirming angiostrongylosis, after the modified Baermann suggested a diagnosis of *Filaroides*. Of the animals with one or more false negative test results, 50% (n = 3) had recently received an anthelminthic which had the potential to decrease their parasitic burden, making a subsequent diagnosis challenging. Modified Baermann was not performed in two animals including one that died before the test was performed and one other which was positive on zinc sulphate flotation. Details of those animals with one or more negative results and the anthelmintic they had received are presented in Table 5.

**Table 5:** Diagnostic parasitological test results from cases negative by one or more methodologies

Case	MB	BAL	PCR	PM	Recent Anthelmintics
**9467**	- ve*	-	-	+ve	none

**33248**	- ve*	- veΩ	-	+ve	none

**34974**	+ ve	-veΩ	-	-	-

**39262**	- ve *	-ve Ω	-	+ve	none

**45801**	- ve	+ ve	-	-	Ivermectin 5 days prior to referral

**52562**	- ve	-	-	+ve	Selamectin 2 and 6 weeks prior to referral

**36236**	- ve*^¥^	-ve *	-	-	-

**61218**	- ve#	-ve	+ve	-	Fenbendazole immediately prior to referral

*: Serial negative on MB or BAL, #: Filaroides suspected on MB, Ω: Cytological evidence of intrapulmonary haemmorhage following BAL, ¥: Tested positive by MB after 10 months

-ve: Negative

+ve: Positive

MB: Modified Baermann

PCR: Polymerase chain reaction

PM: Post Mortem

BAL: Bronchoalveolar lavage

Four (16.7%) animals died before or within 24 hours of instituting treatment. The median age of these animals was six months. All presented with or developed severe dyspnoea with generalised alveolar or alveolar-interstitial patterns on thoracic radiography. Three cases had clinical and clinicopathological evidence of a bleeding diathesis.

A course of fenbendazole (50 mg/kg PO q 24 hours for 5-35 days, median (SIR) 10 (7.25-10) days) was administered in 19 cases. There was no significant response in one (5%) animal that was subsequently euthanased, confirming the previously suspected diagnosis of angiostrongylosis. This dog was older (144 months) and had a myriad diagnoses (arthritis, lymphocytic-plasmacytic enteritis and glomerulonephritis), angiostrongylosis at post mortem. Another older animal was euthanised without treatment after two negative modified Baermann's and again a variety of other pathologies were evident at post mortem. Eighteen (95%) animals responded with a full recovery. Modified Baermann was performed after treatment (median 10 days) in 10 previously positive cases (56%) all of which proved negative. One of these cases presented as a young animal with a strong suspicion of angiostrongylosis but with negative results on numerous modified Baermann flotations and BALs over a six month period. There was initially a limited response to a full course of fenbendazole (50 mg/kg PO q 24 h for 10 days), but 10 months later following a positive diagnosis by modified Baermann a full response to a prolonged (35 day) course was recorded. Two recovered animals had recurrence of similar clinical signs at a later date, neither had a parasitological diagnosis but there was a full response to appropriate treatment.

**Figure 3:**
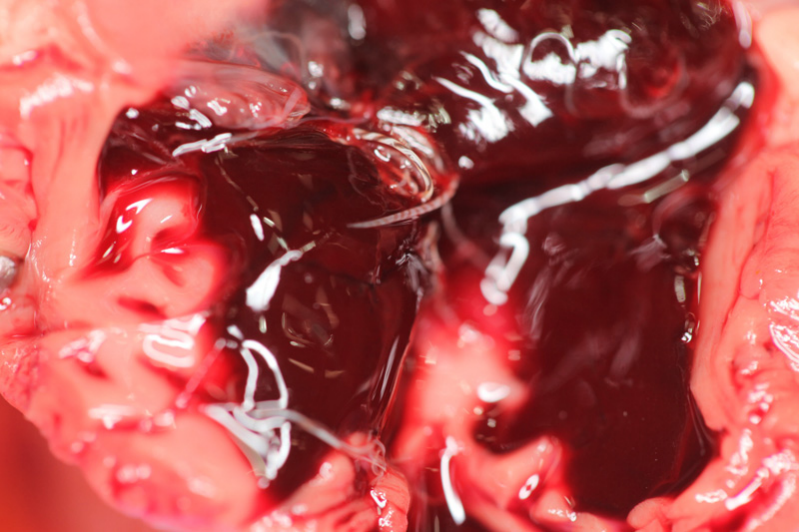
**Diffuse haemorrhagic consolidation of infected lungs**.

**Figure 4:**
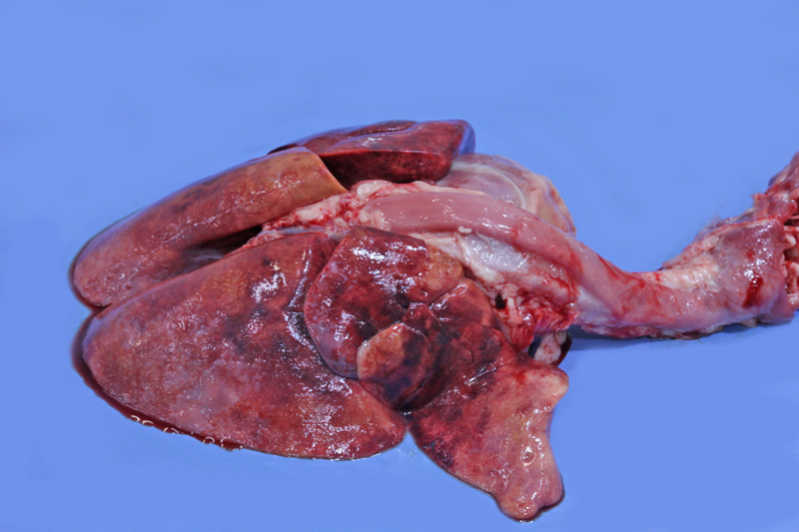
**Adult female (Barber's pole) within the heart**.

Post mortem examination was performed in all cases that died (n = 4) or were euthanased (n = 2). A bleeding diathesis was identified in five animals including pulmonary congestion (n = 4) (Figure 3), injection site subcutaneous haemorrhage (n = 2) and haemorrhagic pleural effusion (n = 1). Cardiopulmonary pathology was frequently noted including gross dilation or hypertrophy of the right ventricle (n = 3), granulomatous pneumonia (n = 2) and pulmonary fibrosis (n = 1). Larvae and/or adults were identified in the right heart or lungs of all six cases (Figure 4). One animal (in which an antemortem diagnosis was not made) had large numbers of nematodes only within pneumonic areas of pulmonary tissue.

## Discussion

The present study illustrates that infection with *A. vasorum *remains endemic in Ireland, is not confined to the East Coast, can occur in any age of dog and has a widely varying clinical picture.

In the present study, approximately 90% of affected animals either directly referred or from external post mortem or faecal submissions were from the East Coast of Ireland. These results may imply an endemic focus in this area. However, the two major counties represented are within the referral catchment area for the UCD Veterinary Hospital, likely creating a degree of bias. Only five animals were from beyond this area with two from the West of Ireland. The only other published report of dogs affected in the West of Ireland was documented decades ago [6]. This may truly reflect a low prevalence beyond the East Coast. However, it could also reflect limited referral of cases from this geographic location or a failure to consider the differential because of a perception that angiostrongylosis does not exist there. Nevertheless, worldwide there is evidence of area expansion, as demonstrated by a recent surge in sporadic cases in non-endemic areas and countries previously considered disease free [13,20,21]. The fact that a diagnosis was made from various different areas of Ireland may support such expansion and suggests that a diagnosis cannot be excluded based on geographic location alone.

Angiostrongylosis is considered to be a disease of primarily young animals. In three separate reports of eight, 23 and 160 dogs, reported ages were less than 24 months (100%), a median of ten months and one year of age or younger (50%)[7,10,22]. The reason why older dogs are less frequently affected are unclear and the possible role of age resistance and immunity requires further study. The median age of 20 months in the present study largely concurs with published data, with 75% of animals diagnosed at less than 2.5 years old. However, while young age appears to be a predisposing factor, angiostrongylosis was diagnosed in this study in dogs of all ages, with a quarter of animals aged between 5 and 12 years, in keeping with other studies [7]. Infection in older dogs could reflect a degree of immunocompromise predisposing to infection. Whatever the reason, this emphasises that a diagnosis of angiostrongylosis should be considered in all dogs with appropriate clinical signs, regardless of age. However, as expected based on a pre-patent period of 49-60 days, it is an unlikely differential in dogs two to three months of age and younger. The youngest referred clinical case in this case series was four months old.

Foxes are a known reservoir for infection. In the UK, the overall prevalence in the fox population is 7.3%, with absence of infection in foxes up to 2006 in northern England and Scotland [23], areas where canine angiostrongylosis was previously absent. However, five cases have by now been reported from these latter areas and may suggest that foxes are currently infected there [12,13]. The fact that unrelated housemates were known to be infected in this series exemplifies shared environmental risk factors. Many owners did report exposure to foxes and ingestion of snails but some did not. Further study of infection in Irish foxes and other potential hosts is therefore warranted.

Of the reported clinical features seen in naturally occurring angiostrongylosis, respiratory compromise is common. Cough and dyspnoea were reported in 65% and 43% of dogs, respectively, in one recent report of 23 cases [7], and in over 50% of dogs in two other series of eight and seven cases [8,10]. Cardiorespiratory compromise was a common complaint in the current study, with cough, dyspnoea and tachypnoea frequently reported. Despite demonstrating marked pulmonary pathology in experimentally infected animals, clinical evidence of respiratory compromise tended to be mild [24]. Similarly in over one third of animals in the current series radiographic evidence of pulmonary pathology ranged from mild to moderate, despite presenting with no overt respiratory signs. The absence of respiratory signs therefore should not preclude consideration of angiostrongylosis as a differential diagnosis. Equally the value of thoracic radiography in investigating angiostrongylosis in the absence of cardiorespiratory signs should not be underestimated.

Although poorly understood, an association between a bleeding diatheses and angiostrongylosis has been firmly established and was the second most common clinical sign observed in 35% of cases [7]. Isolated case reports have described a varied spectrum of haemostatic defects including haemoabdomen [25,26], multifocal brain and spinal cord haemorrhage [27,28], gastrointestinal haemorrhage [7], pleural effusion [17,29] and ecchymotic and ocular haemorrhage [7,13]. Coagulopathy was the most common clinical feature of the current series occurring in approximately twice as many cases as reported previously [7]. This higher proportion may reflect a greater likelihood of practitioners referring coagulopathic dogs, rather than those with less severely critical signs. Clinical signs of the coagulopathy tended to vary widely in both location and severity. As a consequence, specific investigation for angiostrongylosis is prudent in all animals presenting with unexplained haemorrhage.

Neurological signs secondary to *A. vasorum *are frequently reported but until recently infrequently investigated. The aetiology was presumed secondary to ischaemic (thrombosis or arterial emboli) or haemorrhagic insult to the central nervous system, with the latter reported more commonly [27,28,30]. One of two neurologically affected animals presenting in the current case series had multifocal brain haemorrhage demonstrated both on computerisedtomography and post mortem examination [16]. The other recovered with symptomatic therapy (fenbendazole) leaving the mechanism of neurological signs undetermined. As investigation of neurological disease can be both invasive and expensive and considering the breath of neurological derangements described secondary to angiostrongylosis, investigation of and treatment for *A. vasorum *should be encouraged as it involves minimal expense and has the potential to achieve a favourable outcome.

A serendipitous diagnosis is possible in dogs with patent infection but in apparent good health [8,10]. In the present series angiostrongylosis was considered an incidental finding in two cases. One was being investigated for exercise induced collapse already described in its familial breeding line, while another case presented for cough and exercise intolerance. In the latter case clinical, clinicopathological and post mortem findings identified a variety of other pathologies (hypothyroidism, arthritis, laryngeal paralysis, pulmonary fibrosis and angiostrongylosis) likely also contributing to the presenting history. Another animal displayed clinical and radiographic evidence of severe pulmonary pathology, but never progressed to haemorrhagic diathesis, severe neurological signs or death despite the diagnosis remaining undetermined and largely untreated for almost one year. These cases may imply a degree of immunotolerance in some animals or in the former cases represent recent infection not yet fully established.

A perception exists that parasitism commonly induces eosinophilia and this was the most common (39%) haematological alteration reported elsewhere [7]. By contrast it was less frequently reported in the current cases series, although when observed tended to be marked. There were no notably significant serum biochemical changes. Dysregulated macrophage production of 1,25 dihydroxycholecalciferol is the proposed mechanism for hypercalcaemia and hyperphosphataemia in humans with granulomatous disease and has been suspected in three confirmed cases of angiostrongylosis [31]. Only two animals were hypercalcaemic in the current study but both were considered mild. Measurement of vitamin D or its metabolites were not carried out but were unlikely to be involved in one case with a concurrent hypophosphatemia. In conclusion there were no pathogonomic haematological or biochemical changes which were suggestive of angiostrongylosis in either this case series or in other reports of naturally infected animals [7]. Certainly the absence of eosinophilia should not preclude consideration of angiostrongylosis. This finding is supported by experimental studies which despite fulminant disease demonstrate only minor alterations [32,33].

The stimulus for coagulopathic abnormalities and subsequent bleeding diathesis is unclear. Transient alterations in thrombocyte counts, PT, APTT, and Factor V and Factor VII concentrations are correlated in experimental studies to stages of parasite development or host inflammatory response. Consumptive coagulopathy or immune-mediated platelet destruction have been proposed in both experimental and naturally occurring infections [14,32,34-36] Severe thrombocytopenia was present in four cases in the current case series, of which three had no alterations in PT or APTT, supporting the theory of immune-mediated thrombocyte destruction. A prolonged BMBT was observed in five animals with clinical evidence of coagulopathy with reference interval thrombocyte counts in all cases, and normal PT and APTT in four, implying thrombocytopathy as a likely mechanism. Clinical evidence of haemorrhage (haemoptysis) was seen in three cases despite having no PT, APTT, thrombocyte count and BMBT abnormalities. Unfortunately, d-dimers were rarely assessed and fibrinogen degradation product, fibrinogen concentrations and thromboelastography were not performed, which limits the conclusions that can be drawn from these data. Future studies are required to ascertain the exact cause of bleeding in dogs with angiostrongylosis. However, the current study suggests thrombocytopathy or thrombocytopenia may have a significant role in many of the animals presenting with haemorrhage and that with the tests routinely available for many veterinary practitioners (PT, APTT, thrombocyte count and BMBT), reference interval values cannot exclude the risk of significant haemorrhage in infected dogs.

Interstitial, bronchial and alveolar radiographic patterns are all commonly described while vascular patterns are not in both naturally occurring and experimental infections [17,37]. In the current study over 50% of cases were described as having an alveolar infiltrate. This is lower than previously described [17], differs in being diffuse rather than multifocal or peripheral and may be related to the duration of infection. Experimental studies have demonstrated an association between the duration of infection and the pulmonary pattern observed with alveolar patterns most common at patency (7-9 weeks post infection) which are histologically attributed to haemorrhage and granulomatous inflammation [37]. In the current study, interstitial infiltration was the most commonly observed radiographic feature. Pleural fissures were evident in approximately half of the cases with many having a concurrent interstitial pattern, possibly reflecting chronicity. The progression of a bulla to pneumothorax seen in one case has been described previously [10]. The reasons for the differences in pattern and distribution between this and other studies are unclear. It is potentially related to differences in radiological interpretation but may be suggestive of more prolonged infection in many of the cases seen. Despite radiographic abnormalities being detected, approximately one third had no evidence of cough or dyspnoea, exemplifying that significant lung pathology can be present in the absence of obvious clinical signs.

Modified Baermann is considered the most sensitive, currently available method for diagnosis of L1 larvae in dogs infected with angiostrongylosis [5]. Accuracy for detection of angiostrongylosis by modified Baermann was 100% in one review [7] although negative results occur prior to patency or in animals which intermittently shed. Repeat faecal testing is therefore recommended [8]. BAL also demonstrated good sensitivity in naturally occurring and experimental infections with *A. vasorum *larvae identifying 70% and 100% of cases respectively [7,38]. By contrast in the current series, L1 larvae were only detected by modified Baermann and BAL in 73% and 17% of cases respectively, despite repeat sampling in some cases. Recently both serological and PCR tests have been developed but are either not commercially available or not yet fully validated [39-43]. Thus while the modified Baermann remains the quickest and most sensitive non-invasive diagnostic test available ante-mortem, a negative result does not preclude disease, nor should it discourage a treatment trial.

Fenbendazole was the only anthelmintic used in this retrospective study, with success reported in 95% of cases as reported previously. The dose and duration is largely empirical ranging from 20 mg/kg to 50 mg/kg PO q 24 h for 5-21 days [7,10,44]. Authorised therapies include imidacloprid/moxidectin (Advocate^®^, Bayer) for treatment and prevention and milbemycin oxime (Milbemax^®^, Novartis Animal Healthy) for reducing the level of infection [10,45,46]. Milbemycin oxime (Milbemax^®^, Novartis Animal Healthy) requires increased frequency (q 7 d for four weeks) of the standard dose (0.5 mg/kg) but has a comparable efficacy to fenbendazole [45]. Fenbendazole and imidacloprid/moxidectin have no significant difference in efficacy, radiographic features or side effects when administered to mild to moderately naturally infected animals [44].

A mortality rate of 25% was reported in this study, this is similar to the combined mortality of three separate UK case series [7,8,10], but does appear to be unusually high when compared with two large case series in isolation of 2 and 13% [7,44]. Four animals which died within 24 hours had severe dyspnoea similar to another report [7]. Two animals were euthanized because of either failure to confirm a diagnosis or to adequately treat. The difference in mortality reported in various studies is likely to be associated with numerous factors including, the duration of clinical signs prior to referral, the severity of the population affected, the presence or absence of typical clinical signs and the ability to make a prompt diagnosis or provide adequate treatment. It is unlikely to be associated with the treatment course selected as it was similar in all three reports [7,44]. If *A. vasorum *is highly suspected an appropriate therapeutic trial is advised, as negative diagnostic tests do not preclude infection.

In conclusion, angiostrongylosis is a recognised parasitic condition in Irish dogs. A diagnosis should be considered in dogs from all areas and of all ages. The clinical presentation can be variable but should be considered particularly in dogs with clinical or radiographic respiratory and/or coagulopathic signs. A bleeding diathesis was the most common feature and may be present despite normal coagulation. The routinely used parasitological tests can be repeatedly negative. While the outcome is favourable in the majority of animals, a more guarded prognosis is advised for those animals which present with severe dyspnoea, or in which a diagnosis is challenging or the response to therapy is incomplete. Further research on diagnostic tests is required.

## Endnotes

Photographs courtesy of Brian Cloak in the Veterinary Pathobiology Section in the UCD Veterinary Hospital

## Competing interests

The authors declare that they have no competing interests.

## Authors' contributions

BG: Interrogated the case records, completed follow-ups and drafted the manuscript. SB: Interrogated the case records. MZ: Individually assessed the radiographic features of each case. CTM: Supervised the study and drafted the manuscript. All authors read and approved the final manuscript.
